# Transforming hemodialysis access in Kenya: a national assessment of the Managed Equipment Services model

**DOI:** 10.1186/s12913-026-15127-4

**Published:** 2026-07-18

**Authors:** Ephantus Njagi, Sasha Wawira, Jonah Onentiah, Kenneth Iloka, Laban Thiga, Nicholas Muraguri

**Affiliations:** 1 Department of Human Pathology, University of Nairobi, P.O. Box 19676, Nairobi, 00202 Kenya; 2 Independent Researcher, Nairobi, Kenya; 3 Ministry of Health, Nairobi, Kenya; 4 School of Engineering and Technology, Kenyatta University, Nairobi, Kenya

**Keywords:** Managed equipment services, Chronic kidney disease, Hemodialysis, Public-private partnership, Healthcare infrastructure, Kenya

## Abstract

**Introduction:**

Kenya introduced the Managed Equipment Services (MES) model in 2015 as a seven-year national contract to address significant gaps in access to hemodialysis, which had previously been available in fewer than ten public hospitals. The MES aimed to expand hemodialysis capacity by centralizing procurement, standardizing equipment, and ensuring maintenance across all 47 counties. This study provides the first national evaluation of hemodialysis performance under the MES model.

**Methods:**

We conducted a multi-year retrospective analysis of 54 public hospitals equipped through the MES, six years after implementation, using data from the MES contract, the MES final survey, NHIF reimbursement schedules, and the 2019 Population Census. Hemodialysis system performance was assessed across four domains, including capacity, utilization, reporting accuracy, and cost efficiency, using descriptive statistics, time series trends, and discrepancy analysis.

**Results:**

A total of 54 hemodialysis centers equipped with 305 machines were established across all 47 counties, resulting in national densities of 1.14 centers and 6.41 machines per million people. Machine density varied more than 30-fold across counties. Between June 2015 and April 2021, 392,497 hemodialysis sessions were conducted, with monthly utilization increasing by 376%, from 2,100 sessions in June 2015 to 10,173 in April 2021. Time-series regression showed a significant upward trend (+75.5 sessions per month, R^2^ = 0.721), with significant effects of year (F = 258.42, *p* < 0.001) and calendar month (F = 5.00, *p* < 0.001), and two periods of acceleration in June 2016 and January 2020.

Facility throughput ranged from 217 to 79,202 sessions, and 63% of hospitals operated at less than 50% of their machine capacity. Machine-logged session counts exceeded register-based counts by more than 30% in 79% of hospitals, with a mean absolute percentage error of 180.41%. County-level utilization ranged from 89 to 4,363 sessions per million people, a 49-fold difference. The MES program reached breakeven in month 70, with a projected return on investment of 19.76% by month 84.

**Conclusion:**

The MES significantly increased Kenya’s public hemodialysis capacity and supported rapid nationwide growth in service use. However, disparities in geography, facilities, and documentation persisted, with many hospitals operating below capacity and inconsistent reporting. Although the program broke even financially and had a positive return on investment, facility performance varied widely. These results show that expanding infrastructure alone was not enough to achieve equity or efficient hemodialysis service delivery, highlighting the need to improve staffing, data quality, and system coordination to make the most of future renal care investments.

## Introduction

Chronic kidney disease (CKD) is a growing global public health problem, significantly contributing to diseases, deaths, and financial strain on healthcare systems [1]. In 2017, the Global Burden of Disease study estimated that CKD caused 1.2 million deaths worldwide, with projections indicating a continued increase, especially in low- and middle-income countries (LMICs) [2, 3]. The rising prevalence of CKD is mainly driven by aging populations and the increasing rates of diabetes and hypertension, both key risk factors for end-stage renal disease (ESRD) [4–6]. Without early intervention, CKD can progress to ESRD, which requires renal replacement therapy (RRT) for survival. The main RRT options available are hemodialysis, peritoneal dialysis, and kidney transplantation [7, 8]. However, hemodialysis remains the most common treatment globally due to its established infrastructure and the limited availability of kidney transplants in many regions [9].

Although hemodialysis accounts for about 90% of all RRT worldwide, access remains highly unequal [7]. This disparity is predominantly high, especially in LMICs, more so in sub-Saharan Africa, where financial constraints, inadequate infrastructure, and shortages of trained healthcare workers significantly limit service availability [10, 11]. These inequalities create a significant treatment gap for patients with ESRD, with mortality reaching 95–96% for those unable to access hemodialysis and remaining as high as 88% even among those who start treatment [12, 13]. The worldwide trend of limited access and high death rates is especially important for countries facing increasing chronic disease burdens.

Kenya, like many sub-Saharan African countries, has experienced a rapid increase in non-communicable diseases (NCDs) alongside a persistently high burden of infectious diseases [14, 15]. This dual epidemiological challenge has heightened the need for specialized chronic care services, including CKD management. Although national data remain limited, it is estimated that about 3.8–4% of the Kenyan population has CKD, with approximately 0.3–0.4% progressing to ESRD [16, 17]. As a result, the provision of renal care has become increasingly critical for the responsiveness, equity, and sustainability of Kenya’s health system.

Hemodialysis remains the primary form of renal RRT in Kenya, as kidney transplantation, the most effective long‑term treatment for ESRD, continues to be constrained by limited surgical capacity, donor shortages, and high costs [18, 19]. Over time, demand for hemodialysis has been increasing, driven by improved diagnostic capabilities, heightened public awareness, and increased survival among hemodialysis patients [20]. Despite these gains, access had remained highly unequal, particularly in rural and peri-urban areas where 72.5% of the population lives, and health facilities and supporting infrastructure remained underdeveloped [10, 21]. These ongoing disparities highlighted the need to implement systemic reforms to improve equity in specialized care.

Against this backdrop, the Kenyan health system underwent significant reforms over the past 20 years, characterized by decentralization, increased government investment in infrastructure, and steady progress toward universal health coverage (UHC) [22–24]. A key aspect of these reforms was shifting services that were previously focused in urban tertiary centers to county referral hospitals across all 47 counties after devolution [25]. However, despite these structural changes, access to hemodialysis was still very limited before 2015, with only 44 machines available at a small number of major public hospitals, including Kenyatta National Hospital, Moi Teaching and Referral Hospital, Coast General Provincial Hospital, Nakuru Provincial Hospital, and Kisumu General Provincial Hospital, most of which were located in large urban areas [26].

Within this context, the Kenyan government launched the Managed Equipment Services (MES) program in 2015 as a long-term public-private partnership to improve access to specialized health services, including hemodialysis. Under seven-year service contracts with five Original Equipment Manufacturers (OEMs), the program bundled the supply, installation, maintenance, replacement, and user training for medical equipment across the country [27]. Through this model, hemodialysis services were initially established in 49 hospitals and expanded to 54 by 2018, marking the first nationwide effort to provide county-level access to renal replacement therapy that had previously been unavailable.

Six years after the MES rollout, we conducted a nationwide, multi-year retrospective evaluation to assess the impact of the MES model on hemodialysis delivery in Kenya. The evaluation examined service availability, equipment utilization, efficiency, geographic equity, and costs. By providing empirical evidence on how the MES approach has influenced the organization and performance of hemodialysis services over time, this study offers policy-relevant insights into the model’s effectiveness and long-term sustainability, and guides strategies to improve renal replacement therapy in Kenya and other countries with similar health system contexts.

## Methodology

### Study design

This study employed a retrospective, facility-based design to evaluate hemodialysis services provided under MES, a seven‑year contract implemented from June 2015. All 54 MES-supported public hospitals in Kenya were included in the census. The design focused on describing service availability, utilization, and performance during the period immediately preceding contract expiry, with facilities serving as the unit of analysis.

### Settings

Kenya’s health system is decentralized, with service delivery managed by 47 county governments, and the national government overseeing health policy and managing national referral hospitals [25]. The system includes 14,883 health facilities organized into 6 levels. Level 1 consists of community health units. Level 2 comprises dispensaries, medical clinics, and outpatient units, accounting for about 71% of all facilities. Level 3 includes health centers, medical centers, and nursing homes, representing nearly 22% of the total. Levels 4, 5, and 6 offer specialized diagnostic, inpatient, and referral services and together make up approximately 7% of all facilities. Public facilities account for 46% of all health facilities; private, for-profit facilities, 43%; faith-based facilities, 8%; and non-governmental organizations, 3% [28]. Approximately 72.5% of the population lives in rural areas and depends primarily on public facilities for primary health care, as these are often the only health facilities available in such settings [21]. As patients progress through the referral system, the complexity of care increases, supported by higher levels of staff expertise and the availability of specialized equipment, including hemodialysis machines [29].

MES established hemodialysis centers in all 47 counties, mainly at county referral hospitals at Levels 4 and 5, where staffing, infrastructure, and specialized equipment were more available. In nearly all the counties, these MES-supported centers became the only facilities offering hemodialysis services. Understanding this service-delivery structure is essential for understanding how the MES model shaped the expansion and effectiveness of hemodialysis services nationwide.

Health services in Kenya are mainly funded through national and county tax revenues, with extra support from external donors. Providers in the public, private, and faith-based sectors are reimbursed through insurance schemes and other prepayment methods. The National Hospital Insurance Fund (NHIF), Kenya’s mandatory social health insurance scheme, collects member contributions to cover a wide range of inpatient and outpatient services, while private health insurance provides additional coverage for a smaller portion of the population [30, 31]. During the study period, patients requiring hemodialysis usually received two 4-hour sessions per week, each covered by the NHIF at USD 74. Clinicians could suggest a third session weekly if medically necessary, but this additional session would require a personal payment. In 2024, Kenya’s minimum wage ranged from USD 60 per month in rural areas to USD 120 in major cities, while national estimates place median household income between USD 85 and 155 per month, highlighting the significant affordability challenges faced by patients requiring hemodialysis [32].

### Data sources

We utilized data from the MES contract, the final MES survey, facility hemodialysis records, NHIF reimbursement schedules, and the 2019 Kenya Population and Housing Census to evaluate hemodialysis unit and machine density, utilization, reporting accuracy, and cost efficiency. The MES contract provided details on machine site allocation, installation timelines, and contract costs. The final MES survey provided data on machine functionality and operating hours of MES-supplied hemodialysis machines. Facility records provided the number of monthly hemodialysis sessions, while NHIF reimbursement schedules specified the reimbursement rate for hemodialysis services. Population data from the 2019 Census supported calculations of coverage and equity indicators to assess impact.

### Data collection

Data for the MES final survey were collected between May and June 2021. A structured data collection tool was developed to capture dialysis-specific information, including equipment identifiers, installation and commissioning dates, operational status, downtime, and monthly hemodialysis service volumes. No patient-level data were collected. The tool was reviewed by biomedical engineers and clinicians and pre-tested in two non-MES hospitals to ensure clarity and completeness.

A multidisciplinary team of trained health professionals visited all MES-supported hemodialysis units. At each site, the team used a structured questionnaire to collect details about installation dates, functionality, physical condition, and operational status of the hemodialysis machines. During each visit, the team verified equipment identifiers, confirmed operational status, and documented any downtime. Monthly hemodialysis session counts were retrieved from unit records, and machine operating hours were obtained directly from built-in counters. The MES contract cost data were obtained from MES contracts, and the county population estimates were sourced from the 2019 Census. All data points were triangulated from multiple sources, and discrepancies were resolved through consultation with facility staff and review of original documentation.

### Data processing and analysis

Data were entered, cleaned, and coded in IBM SPSS Statistics version 26. Double‑entry checks were performed, and the dataset was examined for missing values, outliers, and logical inconsistencies. Implausible observations were corrected where possible or excluded if unresolved. New indicators were created, including equipment‑to‑population ratios and measures of service utilization. Facility‑level records were aggregated at the county level to calculate machine and unit densities and to visualize geographic disparities in hemodialysis infrastructure.

Key variables included machine availability and functionality, cumulative operating hours, monthly hemodialysis sessions, county population, hemodialysis unit density per 1,000,000 people, hemodialysis machine density per 100,000 people, hemodialysis session rates per 1,000,000 people, and facility-level utilization indices.

The hemodialysis utilization rate was calculated as the number of reported hemodialysis sessions divided by the maximum theoretical capacity and expressed as a percentage. Maximum capacity was estimated based on the number of functional hemodialysis machines, the expected number of sessions per machine per day, and the number of operational days during the reporting period. To standardize potential output across facilities, maximum machine capacity was defined as two sessions per day, five days per week (approximately 43 sessions per month). For each machine, this monthly capacity was multiplied by the number of months it had been operational from installation to April 2021 to derive facility‑level potential capacity, which was then aggregated to the county level. Reported utilization was obtained from hemodialysis registers maintained in each hemodialysis unit and compared with potential capacity to generate utilization rates and a facility‑level utilization index.

Machine-derived utilization was estimated using operational hours recorded on each machine’s internal counter from installation through April 2021. Assuming a standard 4-hour hemodialysis session, total recorded hours were divided by 4 to estimate the number of sessions delivered. Machine-level estimates were aggregated to the facility level to quantify calculated actual hemodialysis output, which was compared with register-reported MES session counts to evaluate reporting accuracy. Differences were expressed as absolute and percentage deviations from machine totals. Variations of 0 to 30% were attributed to routine non-treatment activities such as priming, rinsing, and disinfection that machines record but facilities do not log in registers. Discrepancies of 30% or more were considered indicative of possible underreporting or data entry errors. These comparisons were used to generate a summary table outlining variation between register-recorded and machine-logged hours.

Temporal utilization patterns were analyzed using register‑derived monthly session counts from June 2015 to April 2021. Descriptive monthly trends were plotted to show changes in service delivery over time. To measure long‑term utilization trends, several time‑series analyses were performed. First, simple linear regression estimated the average monthly change in utilization. Second, segmented (piecewise) regression identified statistically significant inflection points in utilization trends, with breakpoints determined using standard structural change detection methods. Third, seasonal variation was evaluated using classical additive decomposition to determine the proportion of utilization variation attributable to seasonal patterns. Lastly, a two‑way analysis of variance (ANOVA) was conducted to examine the independent effects of calendar year and month on utilization. Outliers were detected with standardized residuals and confirmed through significance testing. Statistical significance was set at *p* < 0.05.

Cost efficiency was assessed using the NHIF reimbursement rate of US$74 per session, which was the applicable tariff during the assessment period, to estimate total revenue over 71 months of the 7-year MES contract. The payback period, defined as the time needed for reimbursements to cover the hemodialysis‑related MES contract cost, was calculated by comparing estimated revenue with the contract value of US $28,692,951. Projected revenue for the remaining contract period was estimated based on the observed mean monthly throughput. All financial estimates were reported in nominal terms, without inflation adjustment, to accurately reflect the values faced by facilities and the government during the MES period.

## Results

### Distribution of hemodialysis centers and machines

**Fig. 1:**
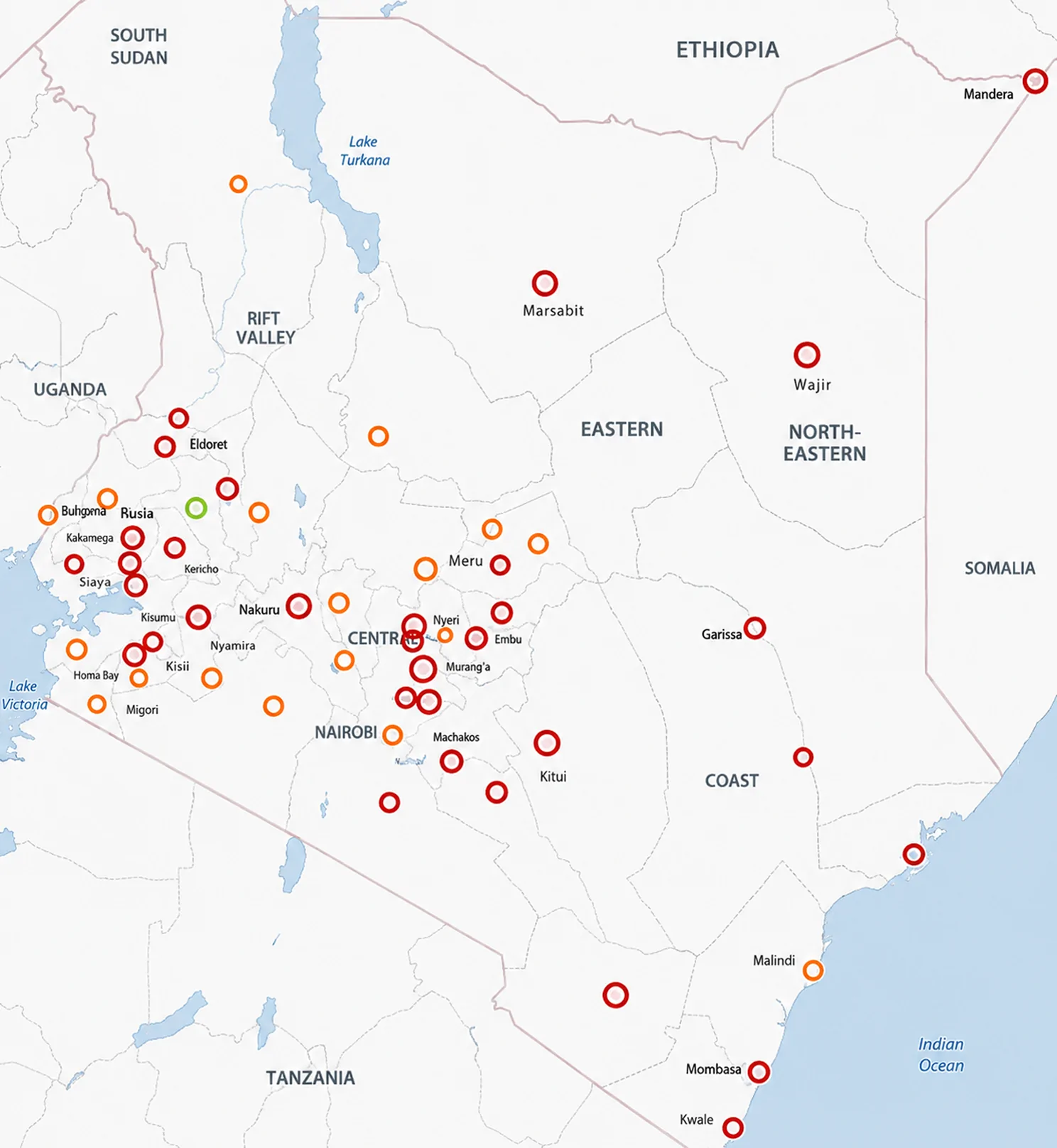
Geographic distribution of hemodialysis centers across Kenya

A total of 54 hemodialysis units were established across 54 hospitals in all 47 counties, resulting in a national density of 1.14 centers per million people (95% CI 1.02–1.26). County‑level densities ranged from 0.45 per million in Nairobi to 6.95 per million in Lamu. Figure 1 shows the spatial distribution of public hemodialysis units across the country.

Across the 54 hospitals, 305 hemodialysis machines were installed, with each facility having 5 to 10 machines, resulting in a national density of 6.41 machines per million people. 31 counties were below this level.

**Fig. 2:**
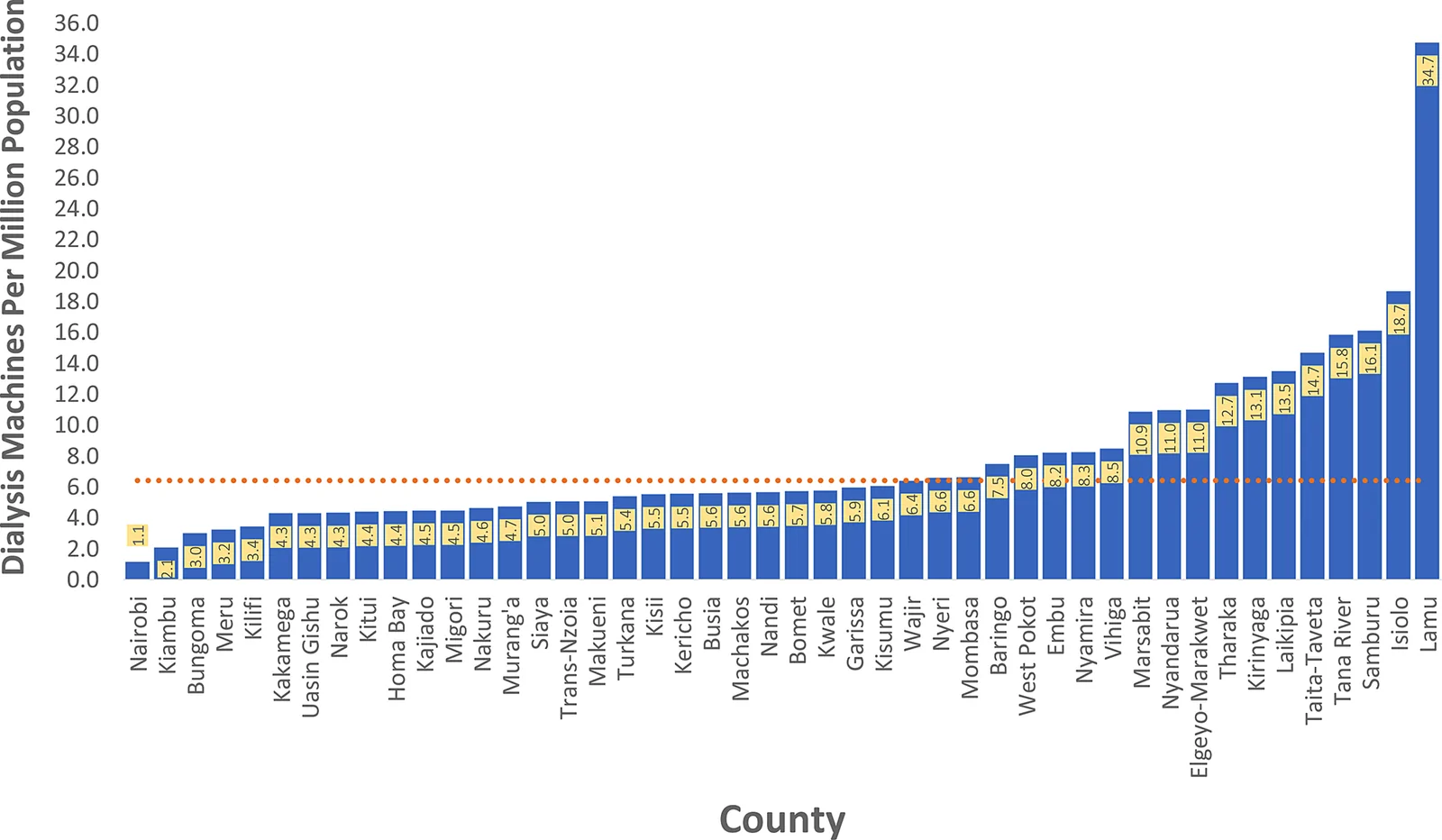
Equity in access to hemodialysis machines across the counties

Machine density varied more than 30‑fold across counties, from 1.1 machines per million (95% CI 0.9–1.3) in the least resourced counties to 34.7 per million (95% CI 33.1–36.3) in the most resourced. Figure 2 illustrates how hemodialysis machine density varies across Kenyan counties, highlighting disparities in the renal care system’s capacity.

### Hemodialysis service utilization

**Fig. 3:**
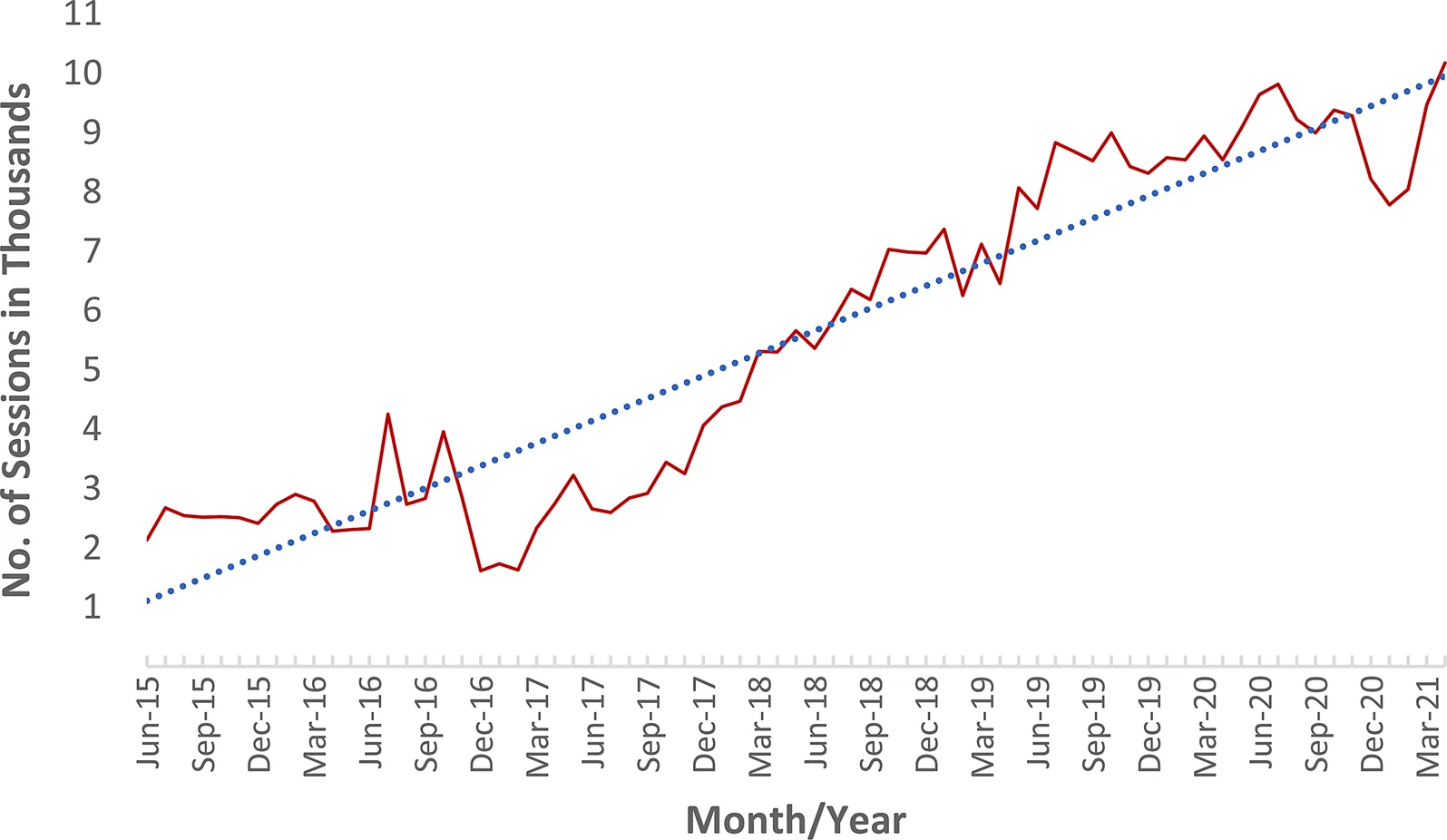
Trends in monthly hemodialysis sessions: June 2015 – April 2021

Between June 2015 and April 2021, the 54 public hemodialysis units delivered a total of 392,497 hemodialysis sessions. The number of sessions rose from 17,312 in 2015 to 108,207 in 2020, a 525% increase. Monthly sessions increased from an average of 2,100 (95% CI 1,980–2,220) in June 2015 to 10,173 (95% CI 9,880– 10,466) in April 2021, a 376% growth. Figure 3 shows monthly hemodialysis sessions from June 2015 to April 2021, with a linear trend line indicating a steady and sustained increase over the period.

Time‑series regression showed a strong upward trend, with sessions increasing by 75.5 per month (95% CI 68.2–82.8; R^2^ = 0.721). Two significant inflection points were identified: in June 2016, the slope increased to 134.8 sessions per month (95% CI 121.4–148.2), and in January 2020, it rose further to 384.2 sessions per month (95% CI 351.7–416.7).

Seasonal decomposition indicated that 33.5% of utilization variance was due to seasonal patterns, with peaks in January–March and June–August. A two‑way ANOVA confirmed significant effects of year (F = 258.42, *p* < 0.001) and calendar month (F = 5.00, *p* < 0.001). December 2016 experienced a sharp decline to 1,612 sessions, a significant outlier (*p* < 0.01), representing a 33% decrease from December 2015. No comparable drops occurred in subsequent years. In contrast, December 2018 recorded 6,966 sessions, and December 2020 had 8,218 sessions, with only a 91-session decrease from December 2019 to December 2020.

Facility throughput varied widely across hospitals, from 217 sessions (95% CI 180 to 254) at the lowest-volume facility to 79,202 sessions (95% CI 78,110 to 80,294) at the highest-volume facility. At the population level, hemodialysis utilization also showed significant variation across counties, ranging from 89 to 4,363 sessions per million people, a more than 49-fold difference. Nationwide, the distribution was right-skewed, with a mean of 1,548, a median of 1,368, and an interquartile range of 1,730 sessions per million.

**Fig. 4:**
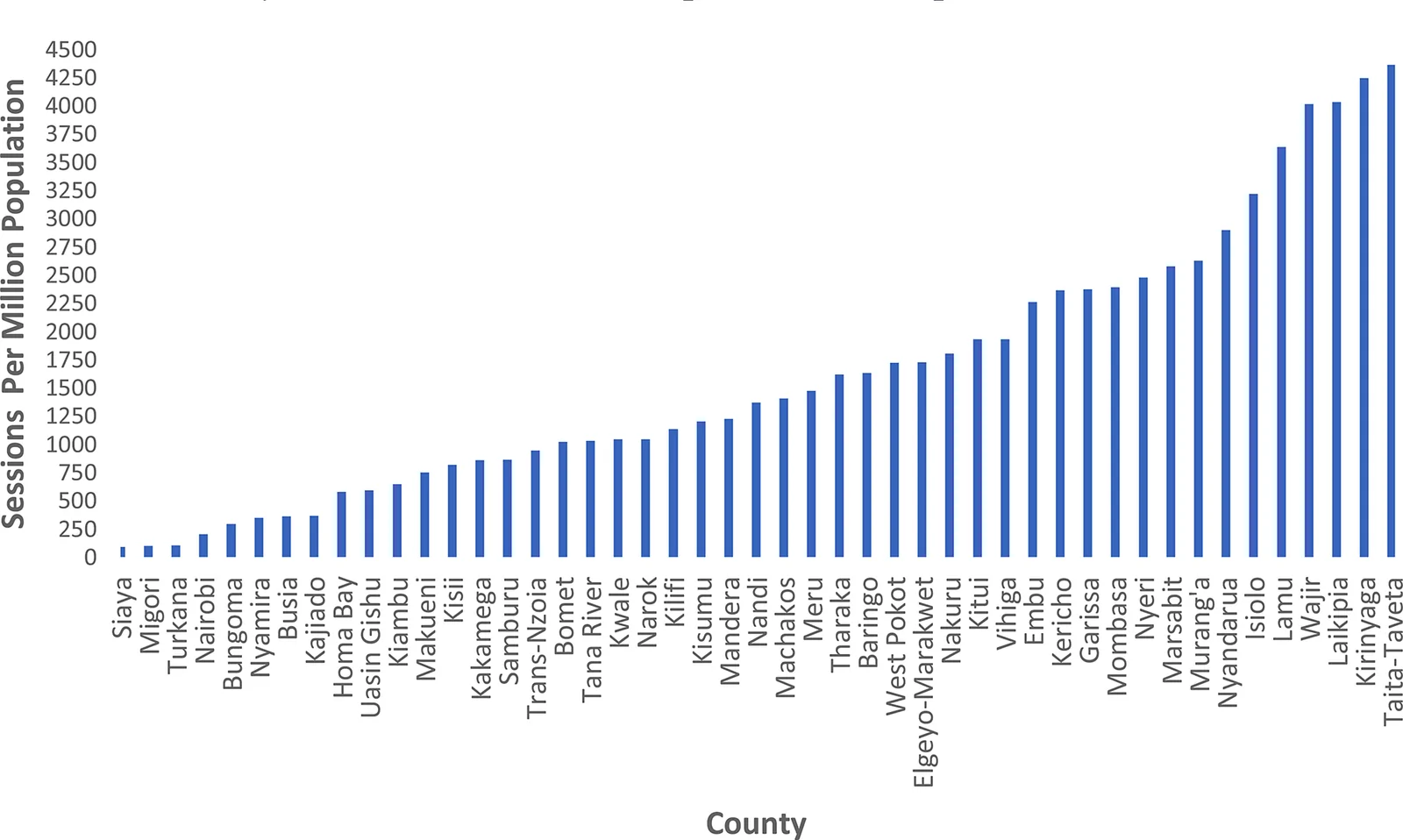
Hemodialysis sessions utilization per million population across counties

As shown in Fig. 4, counties with the highest utilization rates, each exceeding 3,000 sessions per million residents, included Taita Taveta, Kirinyaga, Laikipia, Wajir, Lamu, and Isiolo. Counties with the lowest utilization, all below 600 sessions per million, were Siaya, Migori, Turkana, Nairobi, Bungoma, Nyamira, Busia, Kajiado, and Homa Bay. These findings demonstrate significant geographic variation in hemodialysis service utilization.

### Hemodialysis machine utilization

**Fig. 5:**
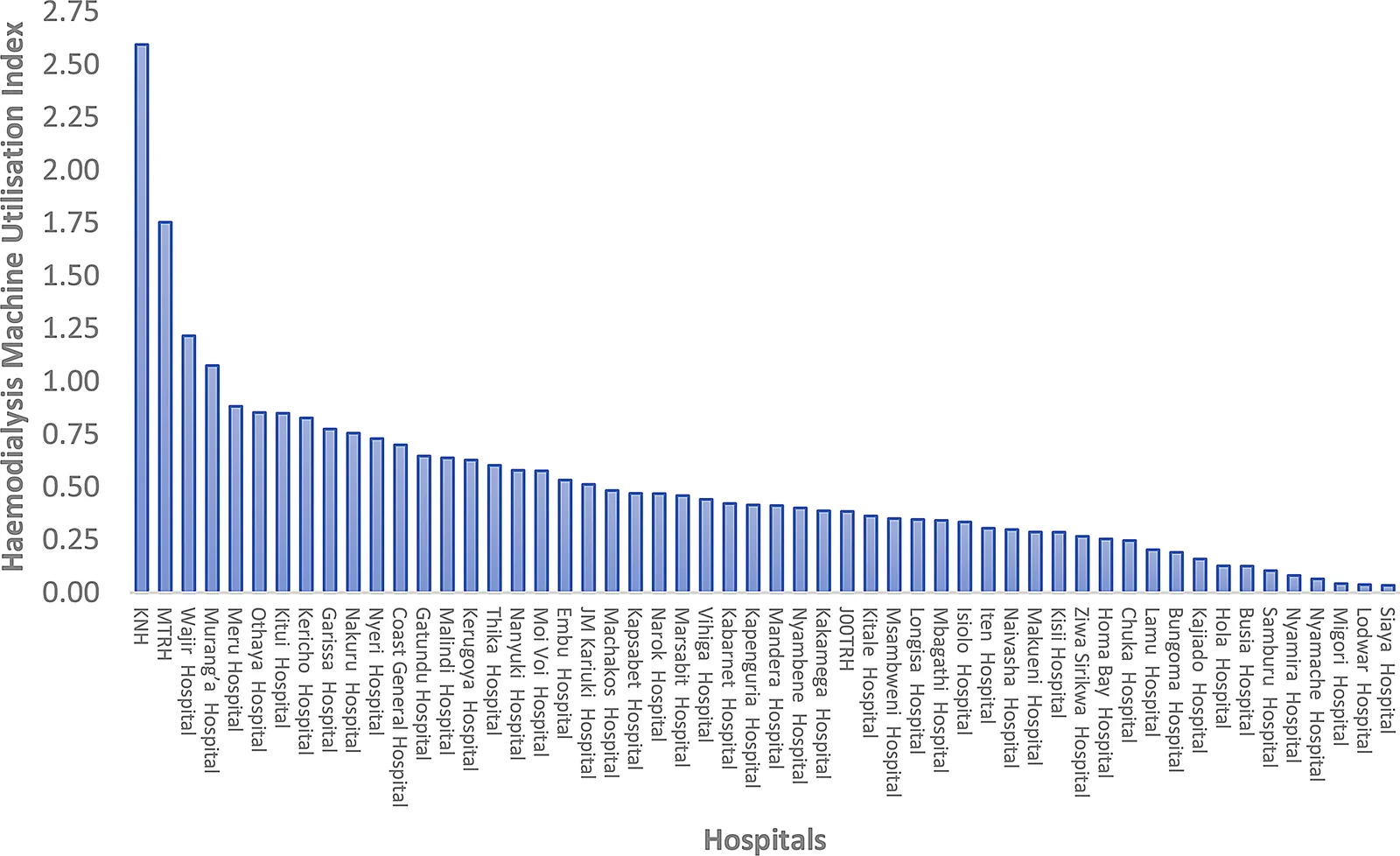
Hemodialysis utilization index across hospitals in Kenya

Machine utilization rates ranged from 0.09 to 1.21, with a median of 0.41 (IQR 0.23–0.58). Overall, 34 hospitals (63%) operated at utilization below 50%, and 11 hospitals (20%) operated at utilization below 25%. Figure 5 illustrates the hemodialysis machine utilization index across public hospitals in Kenya, emphasizing inter-facility differences in service use.

### Discrepancy in hemodialysis session reporting

**Fig. 6:**
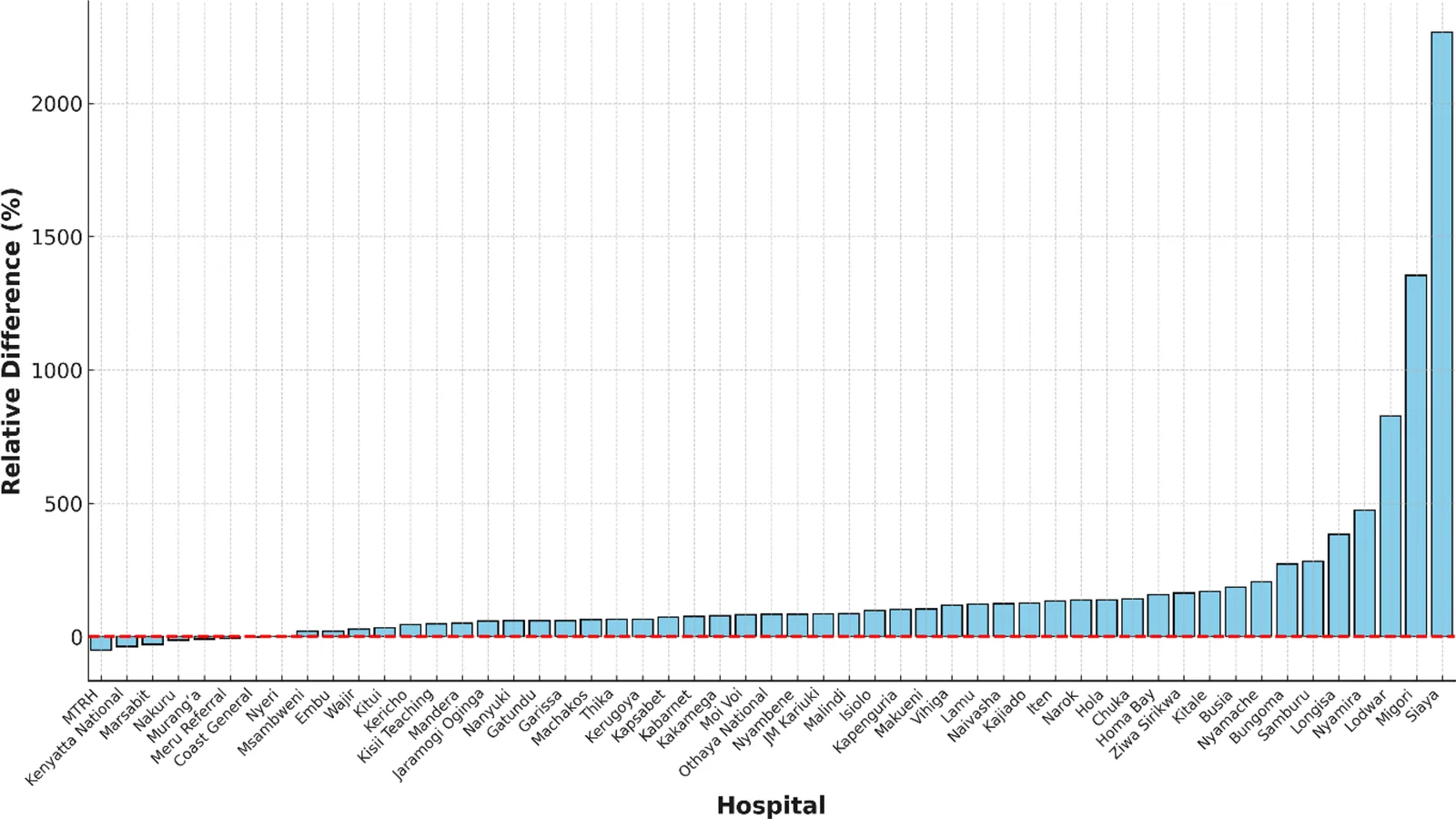
Variation between Register‑Documented and Machine‑Logged hemodialysis Hours

Figure 6 shows the distribution of discrepancies between reported hemodialysis session hours and those recorded by the inbuilt machine counter. Machine‑derived session counts exceeded register‑based counts by more than 30% in 79% of hospitals. The mean absolute percentage error was 180.41% (95% CI 162.8–198.0), and the mean bias was 174.97% (95% CI 158.4–191.5). Discrepancies ranged from 49.79% underreporting to 2,265.65% overreporting, indicating substantial inconsistencies in routine documentation.

### Cost efficiency

From June 2015 to April 2021, the 54 public hemodialysis units in the country performed 392,497 sessions over 71 months. This generated USD 29.05 million in NHIF reimbursements at a rate of $74 per session. The total MES contract cost was $28.69 million, resulting in a net profit of $0.36 million and an interim ROI of 1.24%.

The breakeven point of 387,711 sessions was reached in the 70th month. The observed average monthly throughput was 5,526 sessions (95% CI: 5,214–5,838). Using this throughput, an additional 71,838 sessions (95% CI: 67,782– 75,894) were projected for the remaining 13 months of the contract, amounting to US $5.31 million in additional reimbursements (95% CI: US $5.01–5.61 million).

Over the entire 84-month contract term, total reimbursements were estimated at USD 34.36 million (95% CI: USD 34.06–34.66 million). The projected net return was USD 5.67 million (95% CI: USD 5.37–5.97 million), with a projected ROI of 19.76% (95% CI: 18.7–20.8%). ROI varied substantially across the 54 units. A small subset of six hospitals accounted for nearly half (49%) of all reimbursements, whereas the remaining 48 centers accounted for 51%.

## Discussion

The implementation of the MES, a national PPP model, represented a deliberate attempt to close long-standing gaps in access to hemodialysis services in Kenya. Before its rollout, fewer than 10 public hospitals offered hemodialysis, and annual national output remained below 20,000 sessions, mirroring the broader sub-Saharan pattern of severely constrained access to renal replacement therapy [33–35]. Limited capital investment, inadequate technical capacity, and chronic equipment maintenance failures left most patients with ESKD without treatment, a challenge widely documented across LMIC health systems [18, 36]. The MES aimed to overcome these structural barriers by centrally supplying and installing standardized equipment with guaranteed maintenance, expecting that expanded geographic coverage supported by NHIF reimbursement would increase service use, reduce regional inequities, and strengthen the continuum of renal care in line with bundled procurement and maintenance models [27, 37]. This study provides the first national analysis of whether these intended gains were realized.

The findings show that the MES substantially expanded national hemodialysis capacity, increasing the number of public hemodialysis centers from fewer than 10 to 54, a more than fivefold rise. The national hemodialysis unit density rose to 1.14 per million people, exceeding the sub-Saharan Africa regional average of 0.53 per million [18]. This represents a major structural gain for Kenya’s renal services. However, despite this progress, national density remains well below the global average of 4.46 per million, indicating that the country continues to face a significant capacity gap relative to population‑level hemodialysis needs [20, 38, 39].

Although the MES model expanded hemodialysis capacity across Kenya, the distribution of units and machines remained highly uneven. County-level density varied by more than 15 times, from 0.45 units per million people in Nairobi to 6.95 in Lamu. Machine density ranged from 1.1 to 34.7 per million residents, a 30-fold difference, highlighting the challenges of assigning the same infrastructure to counties with very different population sizes. The results show that universal county coverage did not ensure geographic equity because allocation decisions were based on administrative targets instead of needs-based planning. This pattern aligns with findings from other LMIC settings, where centrally managed procurement caused rapid expansion without adequately addressing epidemiological needs, resulting in a similarly uneven geographic distribution of hemodialysis services [40–43].

Despite geographic disparities, hemodialysis service uptake increased sharply after MES implementation, indicating a large unmet need in Kenya. Annual hemodialysis sessions grew more than sixfold (525%) from 2015 to 2020, while monthly sessions nearly quintupled (376%) from June 2015 to April 2021. This reflects a rapid rise in demand once more hemodialysis capacity became available. These trends indicate that the MES did not just move existing patients between facilities. Instead, it revealed a large pool of unmet needs, with new patients beginning hemodialysis as soon as the machines and units became operational. The scale and immediacy of this growth suggest that many individuals needing hemodialysis were either previously undiagnosed, untreated, or unable to access services due to longstanding barriers such as limited facility availability, high out-of-pocket costs, and geographic concentration of care to the national referral hospitals. The upward trend was systematic rather than random, with the underlying pattern explaining 72.1% of month-to-month variation and showing clear inflection points in June 2016 and January 2020, each roughly a year after major MES rollout milestones. Together, these patterns indicate that increases in physical hemodialysis capacity directly influenced utilization behavior, consistent with scale-up experiences reported elsewhere [44, 45].

Temporal dynamics further influenced utilization. Seasonal decomposition revealed that 33.5% of the variation in monthly sessions was due to predictable seasonal cycles, with peaks from January to March and June to August, and a recurring trough in December. These patterns suggest that utilization is governed not only by clinical need but also by social and economic rhythms that affect care-seeking behavior, consistent with evidence from other chronic care services where holiday-related disruptions, competing household demands, and short-term financial constraints impact attendance [46–49]. The two-way ANOVA confirmed significant effects of both year and calendar month, highlighting the importance of temporal factors in service use.

Not all fluctuations were seasonal. The 33% decline in December 2016 compared to December 2015 coincided with prolonged industrial action by doctors and nurses. Earlier drops in August (35.6%) and November 2016 (28.0%) suggest that system pressures were already building before the peak of industrial action, indicating a gradual erosion of service continuity. A similar significant decline in service utilization across various healthcare systems has been associated with these labor disruptions, reinforcing the notion that the 2016 fluctuations indicated broader system-level instability [50]. These events demonstrate the vulnerability of hemodialysis services to workforce disruptions and the need for strong human resource management [51].

Significant variation also emerged in how facilities used their installed hemodialysis capacity. Although national output rose during the MES period, throughput fluctuated by more than 360 times across hospitals, from 79,202 sessions at Kenyatta National Hospital to just 217 sessions at the lowest-volume facility, showing considerable heterogeneity in operational readiness. Machine utilization rates showed similarly wide disparities, with a median of 0.41, and most hospitals operated at less than half of their installed capacity. In contrast, a small number of high-volume referral hospitals exceeded 100%, suggesting overuse, possibly driven by cross-county patient inflows. At the population level, hemodialysis use varied significantly across counties, with almost a 50-fold difference. Very high per-million utilization in counties such as Taita Taveta, Kirinyaga, Laikipia, Wajir, Lamu, and Isiolo reflected the effects of relatively small population sizes and the lack of private hemodialysis options, which concentrated demand in MES-supported facilities. In contrast, low utilization in large population counties like Nairobi, Kiambu, Bungoma, Busia, and Kajiado persisted despite substantial disease burden, a pattern that reflects both the diversion of patients to numerous private hemodialysis centers in some counties and, in others, the uneven availability of trained dialysis staff and reliable consumables in MES facilities, which limited their ability to provide consistent services and reduced the overall system impact of the MES investment. Collectively, these patterns indicate that infrastructural expansion alone did not guarantee effective or equitable use of hemodialysis services. Maximizing MES capacity requires stronger integration of public and private providers, adequate staffing, reliable maintenance systems, and improved county-level coordination, a pattern also observed in South Africa, where rapid expansion of hemodialysis infrastructure outpaced human resource availability and system readiness [52, 53].

Data quality was identified as a significant limitation. In nearly 80% of hospitals, machine-generated session counts exceeded register-based counts by more than 30%, with an average absolute error of 180.41% and a bias of 174.97%. Discrepancies ranged from 49.79% underreporting to 2265.65% overreporting, indicating systemic issues in facility-level hemodialysis documentation rather than random errors. Poor health record-keeping documentation in hospitals is a well-documented barrier to effective hemodialysis governance in many LMICs [54, 55]. Strengthening digital infrastructure and reducing reliance on manual registers would improve data completeness, enhance accountability, and support more accurate planning for hemodialysis capacity and financing [37, 56].

Financial performance also depended heavily on utilization. The MES broke even around month 70, when cumulative sessions reached 387,711, and by month 71, reimbursements slightly exceeded the contract cost, with US $29.05 million recovered versus US $28.69 million spent on the MES contract. The projected 19.76% return on investment by the end of the 84-month contract indicates that the MES can generate modest financial gains, but only under conditions of consistently high throughput. Nearly half of all sessions were delivered by six hospitals, showing that a small number of high-volume centers drove overall cost efficiency. This pattern aligns with global evidence that hemodialysis services achieve economies of scale only after throughput exceeds specific thresholds, while low-volume facilities struggle to cover operational costs [57–59].

These findings indicate that although MES expanded hemodialysis capacity nationwide, it did not guarantee equity in the distribution of hemodialysis services or optimal utilization of installed capacity across counties and hospitals. In Kenya, ongoing disparities at the county level suggest that universal coverage through equipment allocation and maintenance did not achieve geographic equity, as counties differed greatly in their ability to adopt and operate the technology. Variations in human resources, referral coordination, managerial skills, and the structure of local renal markets, especially the dominance of private providers in urban areas, influenced how well MES units integrated into regular public health services. These structural differences reveal deeper issues with governance and system readiness under devolution, where counties with stronger institutional capacity were better able to turn available, well-maintained equipment into effective services. The evidence highlights the need for planning approaches that match deployment with epidemiological needs, improve workforce and information systems, and strengthen services where economies of scale are possible. Overall, Kenya’s experience shows that expanding technology, even with maintenance requirements, is not enough to ensure equity without parallel investments in governance, maintenance oversight, and coordinated referral networks.

### Limitations

This study has several limitations. First, it relied on facility‑level data and could not incorporate patient‑level characteristics such as comorbidities or socioeconomic status, which limits the ability to determine whether utilization patterns reflect need or facility performance. Second, private hemodialysis centers were excluded, limiting generalizability to the national hemodialysis landscape. Third, key contextual factors, such as staffing levels, supply chain reliability, and county financing decisions, could not be fully assessed, although they are likely to influence utilization and efficiency. Fourth, the cost analysis depended on recorded hemodialysis sessions and NHIF reimbursements, which are vulnerable to documentation errors and do not capture indirect costs, potentially underestimating the true cost of service delivery. Fifth, the study period coincided with major system shocks, including the COVID-19 pandemic and nationwide health worker strikes in 2017. These included the doctors’ strike from December 2016 to March 2017 and the nurses’ strike from June to November 2017. Both events disrupted service availability and altered health-seeking behavior, potentially influencing utilization patterns and, consequently, the estimated return on investment. Finally, the analysis focused on utilization and capacity rather than clinical outcomes and, therefore, cannot determine the impact of MES expansion on survival, quality of care, or long‑term health outcomes.

However, overall, this analysis highlights the complex relationship between infrastructure deployment, health system preparedness, and utilization trends in determining the performance of hemodialysis services.

## Conclusion

The MES significantly increased hemodialysis capacity in Kenya and enabled rapid growth in service utilization, but ongoing geographic disparities, inconsistent facility readiness, and data quality issues limited these improvements. Equipment placement often did not align with population needs, leaving many centers operating well below capacity. At the same time, a few high-volume facilities handled most of the national output and maintained cost efficiency. These structural imbalances were further exacerbated by seasonal service variations and incomplete or inaccurate data, hindering the program’s ability to deliver reliable and equitable care. Solving these problems will require needs-based equipment allocation, better digital recording of machine data, and consolidating services to benefit from economies of scale. A more coordinated and strategic approach is crucial to ensure that increased capacity results in equity, efficient, and sustainable access to renal replacement therapy. As African countries and other resource-limited settings expand hemodialysis services, aligning infrastructure development with population needs, system capacity, and strong governance will be vital for achieving sustainable, high-quality, and equitable kidney care.

## Data Availability

The aggregated datasets used in this study are available from the corresponding author upon reasonable request. All shared data will be fully de‑identified to ensure confidentiality.

## References

[1] Francis A, Harhay MN, Ong ACM, Tummalapalli SL, Ortiz A, Fogo AB, et al. Chronic kidney disease and the global public health agenda: an international consensus. Nat Rev Nephrol. 2024;20(7):473–85. 10.1038/s41581-024-00820-6. 38570631

[2] Bikbov B, Purcell C, Levey AS, Smith M, Abdoli A, Abebe M, et al. Global, regional, and national burden of chronic kidney disease, 1990-2017. A systematic analysis for the global burden of disease study 2017. Lancet. 2020;395. 10.1016/S0140-6736(20)30045-3. PMC7049905 32061315

[3] George C, Mogueo A, Okpechi I, Echouffo-Tcheugui JB, Kengne AP. Chronic kidney disease in low-income to middle-income countries: the case for increased screening. BMJ Glob Health. 2017, 2;2(2):e000256. 10.1136/bmjgh-2016-000256. PMC5584488 29081996

[4] Webster AC, Nagler E V, Morton RL, Masson P. Chronic kidney disease. Lancet. 2017;389(10075):1238–52. 10.1016/S0140-6736(16)32064-5. 27887750

[5] Jha V, Garcia-Garcia G, Iseki K, Li Z, Naicker S, Plattner B, et al. Chronic kidney disease: global dimension and perspectives. Lancet. 2013;382(9888):260–72. 10.1016/S0140-6736(13)60687-X. 23727169

[6] Neuen BL, Chadban SJ, Demaio AR, Johnson DW, Perkovic V. Chronic kidney disease and the global NCDs agenda. BMJ Glob Health. 2017, 2;2(2):e000380. 10.1136/bmjgh-2017-000380. PMC5717948 29225940

[7] Crews DC, Bello AK, Saadi SG. Burden, access, and disparities in kidney disease. Braz J Med Biol Res. 2019, 52;52(3). 10.1590/1414-431x20198338. PMC6437937 30916222

[8] Anand S, Bitton A, Gaziano T. The gap between estimated incidence of end-stage renal disease and use of therapy. PLoS One. 2013;8(8):8. 10.1371/journal.pone.0072860. PMC3758352 24023651

[9] Liyanage T, Ninomiya T, Jha V, Neal B, Patrice HM, Okpechi I, et al. Worldwide access to treatment for end-stage kidney disease: a systematic review. Lancet. 2015;385(9981):1975–82. 10.1016/S0140-6736(14)61601-9. 25777665

[10] Japiong M, Landy CK, Fox MT, Mensah J, Adatara P. Factors affecting access to dialysis for patients with end-stage kidney disease in Sub-Saharan Africa: a scoping review. Nurs Open. 2023;10(10):6724–48. 10.1002/nop2.1970. PMC10495707 37596727

[11] Nyawira L, Tsofa B, Musiega A, Munywoki J, Njuguna RG, Hanson K, et al. Management of human resources for health: implications for health systems efficiency in Kenya. BMC Health Serv Res. 2022;22(1):22. 10.1186/s12913-022-08432-1. PMC9382760 35974324

[12] Ashuntantang G, Osafo C, Olowu WA, Arogundade F, Niang A, Porter J, et al. Outcomes in adults and children with end-stage kidney disease requiring dialysis in sub-saharan Africa: a systematic review. Lancet Global Health. 2017, 5;5(4):e408–17. 10.1016/S2214-109X(17)30057-8. 28229924

[13] Naicker S. End-stage renal disease in Sub-Saharan Africa. Kidney int suppl (2011. Kidney Int Suppl. 2013;3(2):161–63. 10.1038/kisup.2013.4.

[14] Coates MM, Kintu A, Gupta N, Wroe EB, Adler AJ, Kwan GF, et al. Burden of non-communicable diseases from infectious causes in 2017: a modelling study. Lancet Global Health. 2020;8(12):e1489–98. 10.1016/S2214-109X(20)30358-2. PMC8040338 33098769

[15] de-Graft Aikins A, Unwin N, Agyemang C, Allotey P, Campbell C, Arhinful D. Tackling Africa’s chronic disease burden: from the local to the global. Globalization Health. 2010, 6;6(1):5. 10.1186/1744-8603-6-5. PMC2873934 20403167

[16] Muiru AN, Charlebois ED, Balzer LB, Kwarisiima D, Elly A, Black D, et al. The epidemiology of chronic kidney disease (CKD) in rural East Africa: a population-based study. PLoS One. 2020, 15;15(3):e0229649. 10.1371/journal.pone.0229649. PMC7055898 32130245

[17] Mwenda V, Githuku J, Gathecha G, Wambugu BM, Roka ZG, Ong’or WO. Prevalence and factors associated with chronic kidney disease among medical inpatients at the kenyatta national hospital, Kenya, 2018: a cross-sectional study. Pan Afr Med J. 2019, 33;33. 10.11604/pamj.2019.33.321.18114. PMC6815467 31692795

[18] Bello AK, Levin A, Lunney M, Osman MA, Ye F, Ashuntantang GE, et al. Status of care for end stage kidney disease in countries and regions worldwide: international cross sectional survey. BMJ. 2019;367. 10.1136/bmj.l5873. 31672760

[19] Muturi A, Kotecha V, Kanyi S. A retrospective study to assess the evaluation of living related kidney donors and their outcomes following nephrectomy at Kenyatta National hospital. BMC Nephrol. 2017, 18;18(1). 10.1186/s12882-017-0585-7. PMC5445395 28545474

[20] Kabinga SK, McLigeyo SO, Twahir A, Ngigi JN, Wangombe NN, Nyarera DK, et al. Community screening for diabetes, hypertension, nutrition, and kidney disease among kenyans. Kidney Int Rep. 2019, 4;4(10):1482–84. 10.1016/j.ekir.2019.06.015. PMC6829188 31701059

[21] Ministry of health Kenya. Kenya household health expenditure and utilisation survey. Vol. 2014. Nairobi; 2013.

[22] Ministry of Health. Kenya Universal Health Coverage Policy. 2020;2020-2030.

[23] Okech TC, Lelegwe SL. Analysis of universal health coverage and Equity on health Care in Kenya. GJHS. 2015;8(7):8. 10.5539/gjhs.v8n7p218. PMC4965667 26925910

[24] Harris DCH, Davies SJ, Finkelstein FO, Jha V, Donner JA, Abraham G, et al. Increasing access to integrated ESKD care as part of universal health coverage. Kidney Int. 2019;95(4):S1–33. 10.1016/j.kint.2018.12.005. 30904051

[25] National Council for Law. The Constitution of Kenya. Kenya law reports. 2010. 2010, February.

[26] Ndambuki J. The level of patients’ satisfaction and perception on quality of nursing services in the renal unit, Kenyatta National hospital Nairobi, Kenya. OJN. 2013, 03;03(02):186–94. 10.4236/ojn.2013.32025.

[27] Njagi E, Iloka K, Wawira S, Thiga L, Muraguri N. Learning from the Kenyan experiment: key takeaways for implementing managed equipment services in developing countries. Front Health Serv. 2025, 5;5. 10.3389/frhs.2025.1361261. PMC12066563 40356709

[28] Ministry of Health Kenya. Master health facility list: e-health Kenya. http://kmhfl.health.go.ke/. 2022.

[29] Ministry of Health. Kenya health sector referral strategy 2014-2018. Gov Kenya. 2014.

[30] Kazungu JS, Barasa EW. Examining levels, distribution and correlates of health insurance coverage in Kenya. Trop Med Int Health. 2017, 22;22(9):1175–85. 10.1111/tmi.12912. PMC5599961 28627085

[31] Kairu A, Orangi S, Mbuthia B, Ondera J, Ravishankar N, Barasa E. Examining health facility financing in Kenya in the context of devolution. BMC Health Serv Res. 2021, 21;21(1). 10.1186/s12913-021-07123-7. PMC8515645 34645443

[32] Kenya National Bureau of Statistics. Economic Survey. Vol. 2024. Nairobi, Kenya; 2024.

[33] Luyckx VA, Tonelli M, Stanifer JW. The global burden of kidney disease and the sustainable development goals. Bull World Health Organ. 2018;96(6):414–22D. 10.2471/BLT.17.206441. PMC5996218 29904224

[34] Pozo ME, Leow JJ, Groen RS, Kamara TB, Hardy MA, Kushner AL. An overview of renal replacement therapy and health care personnel deficiencies in sub-saharan Africa. Transpl Int. 2012;25(6):652–57. 10.1111/j.1432-2277.2012.01468.x. 22458851

[35] Were AJ, McLigeyo SO. Cost consideration in renal replacement therapy in Kenya. East Afr Med J. 1995;72(1):69–71. 7781562

[36] Sinha A, Bagga A. Maintenance dialysis in developing countries. Pediatr Nephrol. 2015;30(2):211–19. 10.1007/s00467-013-2745-8. 24469439

[37] Geneva: World Health Organization. Global atlas of medical devices. 2017.

[38] Esezobor CI, Alakaloko AE, Admani B, Ellidir R, Nourse P, McCulloch MI. Paediatric nephrology in Africa. Curr Pediatr Rep. 2021;9(4):134–41. 10.1007/s40124-021-00256-7. PMC8542494 34721949

[39] Owino C, Mutugi A, Shah B, Gaffney M, Koech M, Tang J. Global health nephrology education collaboration: a US–kenya experience. Front Med. 2025;12:12. 10.3389/fmed.2025.1647558. PMC12611694 41244762

[40] Jha V, Modi GK. Getting to know the enemy better—the global burden of chronic kidney disease. Kidney Int. 2018;94(3):462–64. 10.1016/j.kint.2018.05.009. 30078513

[41] Moosa MR, Meyers AM, Gottlich E, Naicker S. An effective approach to chronic kidney disease in South Africa. S Afr Med J. 2016;106(2):106. 10.7196/SAMJ.2016.v106i2.9928. 26821893

[42] Maffioli EM, Hernandes Rocha TA, Vivas G, Rosales C, Staton C, Nickenig Vissoci JR. Addressing inequalities in medical workforce distribution: evidence from a quasi-experimental study in Brazil. BMJ Glob Health. 2019;4(6):4. 10.1136/bmjgh-2019-001827. PMC6861089 31798991

[43] Ferraz FHRP, Rodrigues CIS, Sá NMD. Manutenção da iniquidade na oferta do tratamento de diálise crônica no Brasil. Braz. J. Nephrol. 2026;48(1):48. 10.1590/2175-8239-jbn-2025-0033pt.

[44] Kilonzo KG, Jones ESW, Okpechi IG, Wearne N, Barday Z, Swanepoel CR, et al. Disparities in dialysis allocation: an audit from the new South Africa. PLoS One. 2017;12(4):12. 10.1371/journal.pone.0176041. PMC5395209 28419150

[45] Moosa MR, Kidd M. The dangers of rationing dialysis treatment: the dilemma facing a developing country. Kidney Int. 2006;70(6):1107–14. 10.1038/sj.ki.5001750. 16883316

[46] Maeoka Y, Naito T, Irifuku T, Shimizu Y, Ogawa T, Masaki T. Seasonal variation in hemodialysis initiation: a single-center retrospective analysis. PLoS One. 2017;12(6):12. 10.1371/journal.pone.0178967. PMC5456388 28575124

[47] Goto S, Hamano T, Ogata S, Masakane I. Seasonal variations in cause-specific mortality and transition to renal replacement therapy among patients with end-stage renal disease. Sci Rep. 2020;10(1):10. 10.1038/s41598-020-59153-6. PMC7012814 32047207

[48] Argha A, Savkin A, Liaw ST, Celler BG. Effect of seasonal variation on clinical outcome in patients with chronic conditions: analysis of the commonwealth scientific and industrial research organization (csiro) national telehealth trial. JMIR Med Inf. 2018, 20;6(1):e16. 10.2196/medinform.9680. PMC5878365 29549068

[49] Hamad A, Al Halabi AM, Ghazouani H, Habas EM, Borham AM, Ismail SM, et al. Time-series forecasting of hemodialysis population in the state of Qatar by 2030. Qatar Med J. 2023;2023(1). 10.5339/qmj.2023.6. PMC9943994 36846274

[50] Irimu G, Ogero M, Mbevi G, Kariuki C, Gathara D, Akech S, et al. Tackling health professionals’ strikes: an essential part of health system strengthening in Kenya. BMJ Glob Health. 2018, 3;3(6):e001136. 10.1136/bmjgh-2018-001136. PMC6278918 30588346

[51] Thomas S. Building resilience in hemodialysis care: a program report on the british Columbia hemodialysis emergency support team. Can J Kidney Health Dis. 2025;12:12. 10.1177/20543581251363124. PMC12358702 40831907

[52] Etheredge H, Fabian J. Challenges in expanding access to dialysis in South Africa—expensive modalities, cost constraints and human rights. Healthcare (Switz). 2017;5(3):5. 10.3390/healthcare5030038. PMC5618166 28758959

[53] Lee T, Flythe JE, Allon M. Dialysis care around the world: a global perspectives series. Kidney360. 2021, 2. Kidney360. 2021;2(4):604–07. 10.34067/KID.0001082021. PMC8791314 35373050

[54] Nursing documentation: a survey of hemodialysis documentation status at Kenyatta National Hospital’s renal unit. Am Res J Nurs. 2017. 10.21694/2379-2922.17008.

[55] Acharya DK, Nilmanat K, Boonyasopun U. Textual organization of hemodialysis nursing practice: an institutional ethnography. J Nepal Health Res Counc. 2022, 20;20(01):180–85. 10.33314/jnhrc.v20i01.4023. 35945873

[56] Marier-Tétrault E, Bebawi E, Béchard S, Brouillard P, Zuchinali P, Remillard E, et al. Remote patient monitoring and digital therapeutics enhancing the continuum of care in heart failure: nonrandomized pilot study. JMIR Form Res. 2024;8:8. 10.2196/53444. PMC11579625 39504548

[57] Kaur G, Prinja S, Ramachandran R, Malhotra P, Gupta KL, Jha V. Cost of hemodialysis in a public sector tertiary hospital of India. Clin Kidney J. 2018;11(5):726–33. 10.1093/ckj/sfx152. PMC6165756 30288270

[58] Kendix M. Dialysis modality selection among patients attending freestanding dialysis facilities. Health Care Financ Rev. 1997;18. PMC4194467 10175611

[59] Cheng W, Wang H, Yuan J, Li Y, Luo Y, Fu Y, et al. Hospital-based health technology assessment of central dialysis fluid delivery system for hemodialysis patients. BMC Nephrol. 2025, 26;26(1). 10.1186/s12882-025-04484-7. PMC12560572 41146097

